# Variants in *BMP15* Gene Affect Promoter Activity and Litter Size in Gobi Short Tail and Ujimqin Sheep

**DOI:** 10.3390/vetsci12030222

**Published:** 2025-03-02

**Authors:** Shenyuan Wang, Yanyu Bai, Daqing Wang, Ming Zhang, Suhe Alatan, Ming Cang, Hai Jin, Changqing Li, Guangchen Du, Guifang Cao, Bin Tong

**Affiliations:** 1 Inner Mongolia Key Laboratory of Biomanufacture, College of Life Sciences, Inner Mongolia Agriculture University, Hohhot 010020, China; wangshenyuan@imau.edu.cn; 2 The State Key Laboratory of Reproductive Regulation and Breeding of Grassland Livestock, School of Life Sciences, Inner Mongolia University, Hohhot 010020, China; baiyy_imu@163.com (Y.B.); cangming@imu.edu.cn (M.C.); 3 College of Veterinary Medicine, Inner Mongolia Agricultural University, Hohhot 010011, China; wangdaqing050789@126.com (D.W.); duc@imau.edu.cn (G.D.); 4 Inner Mongolia Mengyuan Sheep Breeding Company, Baotou 014016, China; meiligeng777@126.com; 5 East Ujimqin Hexig Animal Husbandry Development Company, Xilingol 026399, China; dwqyzc@163.com; 6 Inner Mongolia Academy of Agricultural and Animal Husbandry Sciences, Hohhot 010031, China; jinhaicnm@vip.sina.com (H.J.); lcqeg@126.com (C.L.)

**Keywords:** *BMP15*, litter size, Gobi short tail sheep, Ujimqin sheep, promoter activity

## Abstract

Reproductive performance in sheep plays a crucial role in determining the economic efficiency of the industry, with increasing litter size being a key focus for genetic improvement. The *BMP15* gene is widely recognized as a major gene influencing sheep fertility. In this study, specific mutations in the *BMP15* gene of Gobi short tail sheep were identified through direct sequencing, and these mutations were genotyped using the MassARRAY system. The g.54285159_54285161TTA indel was significantly associated with litter size in Gobi short tail sheep (*p* < 0.05). Three mutations, including g.54291460G>A, g.54288671C>T, and the g.54285159_54285161TTA indel, were significantly associated with litter size in Ujimqin sheep (*p* < 0.05). Furthermore, the promoter activity analysis demonstrated that the A allele exhibited significantly higher promoter activity compared to the G allele of the g.54291460G>A mutation. These findings highlight valuable genetic markers for improving sheep litter size and provide a robust theoretical foundation for further research on the *BMP15* gene’s role in reproduction.

## Simple Summary

Improving reproductive performance is critical for enhancing the economic efficiency of the sheep industry, with increasing litter size being a primary objective. The *BMP15* gene is a key regulator of sheep fertility. In this study, we analyzed the *BMP15* gene through direct sequencing, and identified three novel mutations, g.54288671C>T, g.54287453C>T, and g.54285159_54285161TTAIndel, and six known mutations, g.54291460G>A, g.54291798C>T, g.54292331G>A, g.54292075C>A, c.755T>C, and c.1047G>A. Association analyses revealed that the g.54285159_54285161TTAindel was significantly associated with the litter size in Gobi short tail sheep. In addition, the mutations g.54291460G>A, g.54288671C>T, and g.54285159_54285161TTAindel were significantly associated with litter size in Ujimqin sheep. Further functional analysis of the g.54291460G>A mutation demonstrated that the A allele exhibited significantly higher promoter activity compared to the G allele, suggesting its potential role in enhancing fertility by increasing *BMP15* expression. These findings identify potential markers for the selection of sheep with improved litter size and provide a foundation for future research on the role of the *BMP15* gene in reproduction.

## 1. Introduction

Sheep are an integral part of the livestock economy in China. Gobi short tail sheep (GB) and Ujimqin Sheep (UM) both belong to the Mongolia (MG) sheep breed [1]. The GB sheep represents an improved breed developed through the selective breeding of MG sheep based on the morphology of a short tail. This breed primarily inhabits arid and semi-arid regions including the Damao, Durbert, and Wulate Banners in western Inner Mongolia. Its distinctive compact caudal structure with reduced adipose deposition aligns with modern consumer preferences for leaner meat while simultaneously lowering husbandry expenses. Comparatively, the UM sheep constitutes an indigenous breed historically distributed across northern China’s steppe regions and southern Mongolia, exhibiting notable biological characteristics including accelerated growth rate, robust vitality, and enhanced environmental stress resistance [2]. However, their small litter sizes limit productivity.

The functional candidate genes are key factors for sheep litter size and ovulation rate [3]. Among them, bone morphogenetic protein 15 (*BMP15*) is globally recognized for its role in regulating reproductive performance [4,5]. Located on the X chromosome, *BMP15* contains two exons and belongs to the TGF-β family [6]. Since research on Romney sheep identified an exon mutation (FecX^I^) associated with increased litter size, the *BMP15* gene has been studied extensively [7]. Variations in *BMP15* have been linked to single nucleotide polymorphisms (SNPs) such as FecX^H^ [7], FecX^G^ [8], FecX^B^ [8], FecX^L^ [9], FecX^Gr^ [10], and FecX^O^ [11]. In addition to SNPs, some variations are not conventional and do not result from a single nucleotide mutation that leads to changes in reproductive traits. For instance, B1 involves a 3 bp deletion in exon 1, while FecX^R^ features a 17 bp insertion–deletion in exon 2 of *BMP15* [8,12,13]. The FecX^Bar^ [11] polymorphism combines single nucleotide substitutions, a 3 bp deletion and a 1 bp insertion, further demonstrating the genetic complexity of *BMP15*, and which have been discovered in various sheep breeds and enhance ovulation rate and litter size. Interestingly, although the majority of homozygous carriers are usually infertile, homozygous high fecundity FecX^Gr^ [10] and FecX^O^ [11] mutations were found in Grivette sheep in France and Olkuska sheep in Poland. Similarly, homozygous high fecundity is present in the FecG^E^ mutation found in Santa Ines sheep in Brazil [14]. However, it has been rarely explored in GB and UM breeds. Thus, identifying GB and UM sheep-specific *BMP15* polymorphisms and unraveling their functional mechanisms are essential for advancing genetic understanding and improving reproductive performance in these breeds.

Among ovine reproductive traits, litter size and ovulation rate significantly impact sheep production, with litter size being the most economically critical [15]. Ovulation rate, which is a prerequisite for litter size, is especially regulated through complex mechanisms [16]. During ovarian development, follicular growth is essential for ovulation and subsequent fertilization [17]. This process involves a close interaction between oocytes and granulosa cells (GCs) [18]. Granulosa cells not only nourish and support oocytes but also produce hormones that promote follicular development and ovulation [19,20]. The apoptosis of granulosa cells is a primary mechanism of follicular atresia [21,22]. Studies on Hu sheep have revealed significant differences in granulosa cell gene expression profiles between individuals with varying reproductive capacities, emphasizing the critical relationship between granulosa cells and oocytes [23]. Similarly, interactions between transcription factors in oocytes and granulosa cells vary across follicular development stages in human ovaries [19]. BMP15 can promote granulosa cell proliferation and differentiation by stimulating mitosis, inhibiting the expression of follicle-stimulating hormone (FSH) receptors, and activating ligand expression [5]. Therefore, research into the gene expression patterns of *BMP15* in granulosa cells is crucial for understanding ovarian development and ovulation.

## 2. Materials and Methods

### 2.1. Sample Selection

The parous ewes of GB (n = 231) and UM (n = 153) sheep aged two years old were used in this study [2]. Both groups of sheep were healthy, of the same age, and consisted of fertile, reproductively mature ewes. Mating occurred naturally without the use of specific rams. Additionally, the sheep were kept under similar environmental conditions, with unrestricted access to food, water, and natural light. Blood samples (10 mL per sheep) were collected from the jugular vein using EDTA-K2 anticoagulant tubes and transported to the laboratory within 24 h. All animal care and experimental procedures adhered to the ethical guidelines set by the Administration of Affairs Concerning Experimental Animals in China. The study was approved by the Institutional Animal Care and Use Ethics Committee of Inner Mongolia University on 15 May 2015 (approval number IMU-2015-03).

### 2.2. DNA Extraction and Sequencing

DNA was extracted from the 384 blood samples using the Tiangen Blood/Cell/Tissue Genomic DNA Extraction Kit (DP304; Tiangen Biotechnology Co., Ltd., Beijing, China) following the manufacturer’s guidelines. DNA quality was assessed by agarose gel electrophoresis and UV spectrophotometry. Twelve pairs of oligonucleotide primers (Table S1) were designed for the PCR amplification of the promoter and exonic regions of the ovine *BMP15* gene using the current X chromosome assembly (ARS-UI_Ramb_v2.0, NCBI RefSeq: NC_056080.1). PCR amplification was performed according to the TAKARA GXL DNA polymerase instructions (R050Q, Takara Bio, Kusatsu, Shiga, Japan). PCR products were analyzed by 3.0% agarose gel electrophoresis to assess quality and quantity, and were subsequently sequenced by Sanger sequencing at Beijing Genomics Institute (BGI, Beijing, China).

### 2.3. SNP Genotyping Using iPLEX MassARRAY

The genotyping of six previously identified variants [24] and three novel ones was performed using the MassARRAY^®^ SNP genotyping system (Agena Bioscience, San Diego, CA, USA) on 231 GB sheep and 153 UM sheep. Primers for *BMP15* PCR and extension were designed with Assay Design Suite v3.0 (https://www.agenabio.com/services/assays-by-agena/, accessed on 5 October 2024), targeting sequences that included each mutation and approximately 100 bases on either side (Table S2). Genotyping was conducted on the Sequenom MassARRAY iPLEX platform, and the resulting data were processed using MassARRAY Typer 4.0 software (Agena Bioscience, San Diego, CA, USA) [25].

### 2.4. Paraffin Sections and Immunohistochemical Staining

The tissues were preserved in 10% neutral buffered formalin for paraffin embedding and immunohistochemical analysis. The protocol for the paraffin sections was as follows: After fixation, the ovary was excised and subjected to a graded ethanol dehydration series, clearing, paraffin infiltration, embedding, and sectioning into 0.5 μm thick slices. The sections were deparaffinized in xylene, stained with hematoxylin for 10 min, followed by eosin for 5 s, cleared, mounted, and examined under a microscope. The protocol for immunohistochemical staining was as follows: After deparaffinization, 5 μm ovarian tissue sections were incubated in a sodium citrate solution (80 °C, pH 6) for 30 min. The sections were then treated with 3% hydrogen peroxide (H_2_O_2_) for 15 min before being incubated overnight at 4 °C with a rabbit polyclonal BMP15 antibody (1:100, Abcam, Cambridge, UK). Following 3 PBS washes, the sections were incubated with DAB for 5 min to develop color, then finally underwent counterstained observation. Control sections, which were not treated with primary antibodies, were included in all experiments.

### 2.5. Plasmid Construction

A 1158 bp fragment of the ovine *BMP15* promoter was amplified and ligated into the pGL4.23 plasmid (Promega, Guangzhou, China) at the *Xho*I and *Bgl*II restriction sites (Thermo Fisher Scientific, Waltham, MA, USA), creating the pGL4.23-Promoter-G and pGL4.23-Promoter-A dual-luciferase reporter constructs. Details of the annealing temperatures used for amplification are provided in Table 1.

### 2.6. Cell Culture and Transfection

The healthy 80 ovaries were freshly harvested at a commercial slaughterhouse, rinsed, and stored in PBS containing penicillin (100 μg/mL) and streptomycin (100 μg/mL) before being transferred to culture dishes. Granulosa cells were isolated by puncturing large antral follicles with hypodermic needles. To evaluate cell purity, the expression of the follicle-stimulating hormone receptor (FSHR), a specific marker for granulosa cells, was assessed. Immunofluorescence analysis showed that over 95% of the isolated cells were FSHR-positive, confirming their high purity and suitability for further experiments (Figure S1).

Granulosa cells were initially seeded in 24-well plates. The cells were then transfected with a mixture of pGL4.23-Promoter-G or pGL4.23-Promoter-A vectors and pGL4.74, combined in a 50:1 ratio. Transfection was carried out using Lipofectamine™ 3000 (Thermo Fisher, Carlsbad, CA, USA), with each well receiving 1 μg of the plasmid mixture. The cells were analyzed 48 h after transfection.

### 2.7. Dual Luciferase Activity Assay

Renilla luciferase served as an internal control. Transfected granulosa cells were collected 48 h after transfection, and luciferase activity was determined using the Dual-Luciferase Reporter Assay System (Promega, Madison, WI, USA) following the manufacturer’s protocol. All experiments were performed in triplicate.

### 2.8. Bioinformatics Analysis

Transcription factor binding motifs within the *BMP15* promoter region were identified using the JASPAR database, https://jaspar.elixir.no (accessed on 15 October 2024).

### 2.9. Statistical Analyses

The parameters of genetic diversity were computed using Excel v16.0, including population genetic indicators, expected heterozygosity (He), observed heterozygosity (Ho), effective allele numbers (n_e_), polymorphism information content (PIC), and the Hardy–Weinberg equilibrium. Linkage disequilibrium (LD) was analyzed with HAPLOVIEW v.4.2 [26]. The frequencies of each mutation allele were assessed using a *χ*^2^ test. A least-squares mean analysis was applied to investigate the SNP’s impact on litter size in the two sheep breeds. The UM population included sheep from this study and a combined population of UM with different variant genotypes from previously published data. The statistical model was defined as follows: Y_ij_ = μ + B_i_ + G_j_ + e_ij_, where Y_ij_ is the phenotypic value of litter size, μ is the population’s overall mean, B_i_ is the fixed effect of breed, G_j_ is the fixed effect of genotype, and e_ij_ is the random error [24]. When fewer than ten sheep exhibited a specific genotype, the reliable evaluation of associations and effects was not possible. Luciferase activity (LUC) was quantified by calculating the ratio of Firefly-to-Renilla luciferase signals. The results are expressed as mean ± SEM.

## 3. Results

### 3.1. Ovine BMP15 SNP Identification in GB Sheep

Nine mutation sites of *BMP15* were identified through direct sequencing. Among these, six sites have been previously reported by our laboratory in MG and UM [24]. These included four mutations in the promoter region (g.54291460G>A, g.54291798C>T, g.54292331G>A, and g.54292075C>A) and two mutations in the exon 2 (c.755T>C (L252P) and c.1047G>A (349V)) of *BMP15*. Additionally, three novel mutations were identified, all located in the intronic regions: g.54288671C>T, g.54287453C>T, and g.54285159_54285161TTAIndel (Figure S2) (NCBI RefSeq: NC_056080.1).

### 3.2. Genetic Diversity Analysis

For each identified SNP, the allele and genotype frequencies, as well as genetic indices (Ho, He, n_e_, and PIC), were analyzed and documented in both GB and UM sheep populations (Table S2). Notably, the g.54287453C>T was exclusively found as the CC genotype in the UM sheep population (Table S3).

### 3.3. Linkage Disequilibrium Analysis of Variants in BMP15

**Figure 1:**
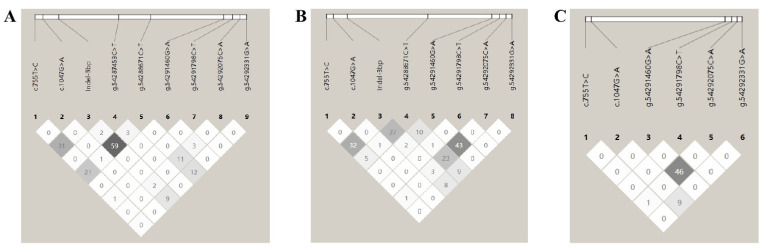
Linkage disequilibrium (LD) estimated among *BMP15* variations in the Gobi short tail sheep and Ujimqin sheep populations. Numbers represent *r*^2^ × 100. (**A**) Gobi short tail sheep. (**B**) Ujimqin sheep in the sample of this study. (**C**) Ujimqin sheep in the combined population of Ujimqin sheep from this study and previously published data. Indel-3bp: g.54285159_54285161TTAindel.

To assess the linkage relationship between the identified mutations, the D’ and *r*^2^ values were computed for two sheep populations. In the GB population, only g.54285159_54285161TTAindel and g.54288671C>T exhibited a moderate linkage disequilibrium, while all other loci showed a low linkage disequilibrium. In the UM sheep, only g.54291460G>A and g.54292075C>A exhibited a moderate linkage disequilibrium, while all other loci showed a low linkage disequilibrium in this study. Additionally, for loci that were previously reported, the current data were combined with earlier findings to assess linkage in a larger UM population. In the combined UM sheep population, a moderate linkage disequilibrium was specifically observed between g.54291460G>A and g.54292075C>A, whereas all other loci pairs showed a low linkage disequilibrium (Figure 1, Tables S4–S6).

### 3.4. Associations Between Novel Variants and Litter Size

#### 3.4.1. Associations Between Novel Variants and Litter Size in Gobi Short Tail Sheep

A total of 231 GB were analyzed in this study, and no FecB mutation was detected [2]. Subsequently, the effects of nine identified variants on litter size were evaluated. The results showed that the TTA.TTA genotype in g.54285159_54285161TTAindel exhibited a significantly higher litter size compared to the DEL.DEL genotype (*p* < 0.05). For other SNPs, no significant association was observed between their genotypes and litter size in GB sheep (Table 2).

#### 3.4.2. Associations Between Novel Variants and Litter Size in Ujimqin Sheep

In this study, 153 UM were analyzed, and no *FecB* mutations were detected. The associations between the identified variants and litter size were examined. For the same variants detected, the data on litter size in UM sheep from this study were integrated with previously published data from our laboratory for further analysis. The results revealed that among the 153 UM sheep analyzed, the GA genotype at the g.54291460G>A site in the promoter region was associated with a significantly higher litter size than the AA genotype (*p* < 0.05). For the g.54285159_54285161TTAindel site, individuals with the TTA.DEL genotype showed a significantly higher litter size than those with the TTA.TTA genotype (*p* < 0.05). Similarly, the CT genotype at the g.54288671C>T was associated with a significantly higher litter size compared to the TT genotype (*p* < 0.05) (Table 3).

When integrated with our previously published data, the individuals with GG at the g.54291460G>A site demonstrated a highly significantly higher litter size than the individuals with GA genotype (*p* < 0.01). Additionally, at the g.54292075C>A site, the CC genotype was associated with a significantly higher litter size than the CA genotype (*p* < 0.05) in the combined UM sheep population (Table 4).

### 3.5. Localization of BMP15 in the Ovary

**Figure 2:**
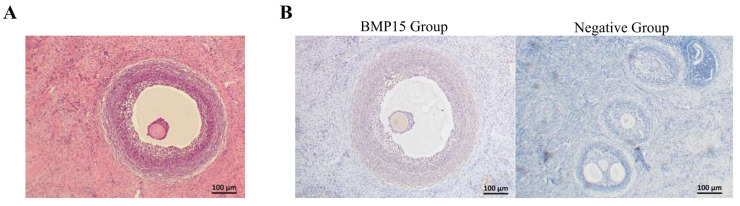
Paraffin-embedded sections of sheep ovaries. (**A**) Histological structure visualized by hematoxylin and eosin (HE) staining. (**B**) Localization of BMP15 protein by immunohistochemical staining, where yellow or gray is indicated as being positive.

To localize the BMP15 protein in sheep ovaries, paraffin sections were prepared and subjected to immunohistochemical staining. The results revealed positive staining primarily in the oocytes, with positive signals detected in the surrounding granulosa cells and follicular wall (Figure 2). These results indicate that *BMP15* is predominantly expressed in oocytes, with granulosa cells adjacent to the oocytes also exhibiting BMP15 protein expression.

### 3.6. The SNP g.54291460G>A in the Promoter of BMP15 Affects Promoter Activity

**Figure 3:**
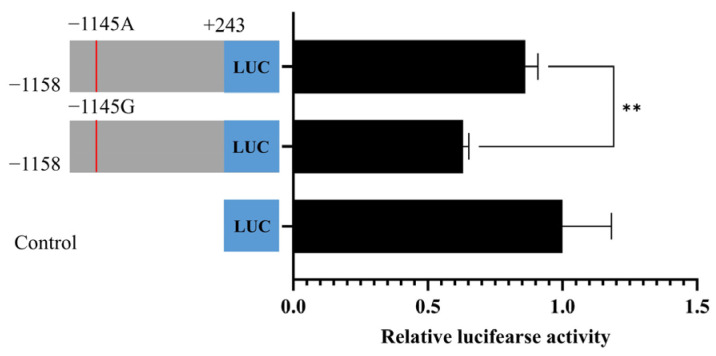
*BMP15* promoter activity analysis in granulosa cells. The g.54291460G>A SNP affects the promoter activity of the *BMP15* gene. **: *p* < 0.01.

Since the g.54291460G>A locus was significantly associated with litter size in both the UM sheep of this study and the combined UM population, we investigated whether g.54291460G>A influenced *BMP15* promoter activity. To this end, a dual-fluorescent reporter vector was constructed using the pGL4.23 plasmid, incorporating either the G or A allele. The results from the dual-luciferase reporter assay showed that the Firefly-to-Renilla luciferase activity ratio was significantly higher for the A allele compared to the G allele of g.54291460G>A (*p* < 0.01) (Figure 3).

### 3.7. Bioinformatics Analysis of Ovine BMP15

Given that the Firefly-to-Renilla luciferase activity ratio was significantly higher for the A allele compared to the G allele in the dual-luciferase reporter assay, we hypothesized that this SNP might alter the transcription factor binding motif of the *BMP15* promoter region. To test this hypothesis, we analyzed a 21 bp sequence surrounding the g.54291460G>A locus of the *BMP15* gene for both alleles. The results revealed that the A allele was associated with the increased binding of four transcription factors. Specifically, the vitamin D receptor (VDR) binding motif was identified as GTTCATACC, the XBP-1 binding motif as GTTCAT, and the FOXP3 and pregnane X receptor-1 (PXR-1) binding motifs as GTTCAT and AAAGTTCA, respectively (Figure S3).

## 4. Discussion

Bone morphogenetic protein 15 (*BMP15*), a significant component of the transforming growth factor-β (TGF-β) family, is crucial for follicular development and ovulation rate as it activates the SMAD1/5/8 signaling pathway through BMP type II receptors and ALK-6. [5,6,27]. Many mutations in *BMP15* play a crucial role in fecundity across different sheep breeds [3], but these mutations have been predominantly observed in European sheep breeds. Our previous reports have shown that the FecX^H^, FecX^I^, FecX^B^, FecX^G^, FecX^L^, FecX^R^, FecX^Bar^, FecX^O^, and FecX^Gr^ mutations of *BMP15* were absent in MG sheep populations [3], and twelve novel and two known c.31_33CTTinsdel (B1) and c.755T>C mutations of *BMP15* were present in the MG and UM sheep breeds [24], together with three novel variants identified in the GB sheep of this study, which showed high breed specificity. Meanwhile, these studies opened new avenues for investigating the association between the *BMP15* gene and litter size in sheep, as well as for exploring the gene’s functional and mechanistic roles.

In association studies, the relationships were not established between the c.755T>C mutation and litter size in GB and UM. This result is consistent with our previous results in UM, as well as the association results of the silent c.1047G>A mutation in the two studies [24]. Nonetheless, predicted amino acid changes at L252P (c.755T>C) were found to impact the BMP15 structure and are linked to litter size in the Afshari, Ghezel, Shal, Xinjiang Cele Black, and MG sheep breeds [24,28,29]. Therefore, the c.755T>C variant may serve as a valuable marker for enhancing litter size across various sheep breeds. In addition to the c.1047G>A and c.755T>C variants, the g.54285159_54285161TTA indel was significantly associated with increased litter size in UM and GB sheep populations. Intronic mutations have the potential to interfere with transcriptional regulatory elements and non-coding RNA genes [30,31]. Given that non-coding sequences constitute the majority of protein-coding genes and are involved in RNA-mediated gene silencing [32,33], it is plausible that the g.54285159_54285161TTA indel affects *BMP15* mRNA splicing or silencing, thereby altering its expression. Although endogenous BMP15-derived miRNAs have not been identified in mammals, a study has reported that a significant association between the human *BMP15* polymorphism rs3897937 and a higher frequency of the A allele was observed in the mothers of spontaneous dizygotic twins [34]. This raises the possibility that the g.54285159_54285161TTA indel may similarly influence *BMP15* expression in sheep, but further research is needed to elucidate its precise mechanism. On the other hand, for the significant association results between the g.54291460G>A SNP and litter size in UM, this result not only provided a potential molecular marker for UM breeding, but also suggested that the impact of the g.54291460G>A site on *BMP15* expression should be researched. In goats, high-yield individuals exhibit significantly elevated *BMP15* mRNA expression in follicles [35], and in cattle, *BMP15* expression in ovarian granulosa cells increases with age, influencing oocyte developmental competence [36]. In our study, we found that the g.54291460G>A variant significantly enhanced the promoter activity of *BMP15*, and ewes belonging to the GA genotype showed significantly higher litter sizes than those belonging to the GG genotype, although the AA genotype was too rare to reach statistical significance. These findings could suggest that high *BMP15* expression may have a stimulatory effect on ovulation and litter size in UM.

Actually, *BMP15* could enhance the expression of anti-Mullerian hormone (AMH) and its specific receptor, anti-Mullerian hormone receptor type 2 (AMHR2), in the granulosa cells of sheep antral follicles by enhancing AMH and AMHR2 promoter activity [37]. AMH is accepted as a remarkable endocrine biomarker in ruminant species. Specifically, *AMH* is expressed in the granulosa cells of growing follicles, and it critically regulates follicle growth and maturation, particularly by inhibiting the FSH sensitivity of follicles [38]. Furthermore, litter size was significantly higher in the high AMH group than in the low AMH group of Romanov sheep [39]. Although, many reports suggested that *BMP15* overexpression inhibits FSH receptor expression, thereby reducing ovulation rate [40,41,42]; meanwhile, many well-characterized *BMP15* mutations have also been linked to increased ovulation rates in sheep [43]. These mutations are typically loss-of-function variants that impair *BMP15* activity, leading to increased ovulation rates in heterozygous animals [43]. However, in the Grivette sheep of France and the Olkuska sheep of Poland, a homozygous mutation with high fecundity was found with FecX^Gr^ and FecX^O^ mutations [10,11]. Together with results of the association and Dual Luciferase Activity Assay of this study, it is speculated that this g.54291460G>A SNP existing in the promoter region promotes the expression levels of the *BMP15* gene, thereby enhancing *AMH* promoter activity, which results in influencing the follicular growth ovulation in UM sheep.

To further explore the molecular mechanism of the *BMP15* g.54291460G>A mutation, we examined its effect on cis-regulatory elements in the promoter region. Our analysis indicated that the A allele introduced a novel binding site for the vitamin D receptor (VDR), a ligand-activated transcription factor linked to reproductive tissue function [44,45]. The VDR heterodimerizes with the retinoid X receptor (RXR) upon ligand binding and acts as a molecular switch in the 1,25(OH)2D3 signaling pathway [46,47]. This potential involvement of VDR in *BMP15* transcriptional regulation suggests a novel mechanism through which the vitamin D signaling pathway may influence ovarian function. Further experimental validation is required to determine whether vitamin D levels interact with *BMP15* expression to regulate litter size.

In summary, this study highlights the critical role of *BMP15* variants in regulating ovulation and litter size in sheep. Specifically, the g.54291460G>A variant enhances *BMP15* promoter activity, which maybe results in creating a novel VDR transcription factor binding site, suggesting that vitamin D signaling may regulate *BMP15* through this mechanism. More in-depth research should validate these variants’ effects on litter size in expanded sheep populations from the GB and UM breeds.

## 5. Conclusions

This study revealed breed-specific responses in the association between genetic variations and litter size. In UM sheep, both in the current sample and the combined dataset from previous studies, the g.54291460G>A mutation consistently showed a significant influence on litter size. Additionally, two newly identified variants, g.54288671C>T and g.54285159_54285161TTAindel, were also found to potentially affect litter size in UM sheep. Moreover, g.54285159_54285161TTAindel was significantly associated with litter size in GB sheep. Functional validation of the g.54291460G>A site demonstrated that the promoter activity of the A allele was significantly higher than that of the G allele. These findings provide novel genetic markers for sheep breeding and offer a theoretical basis for future research on *BMP15* and its role in reproduction.

## Figures and Tables

**Table 1:** Dual-luciferase vector construction primers.

Name	Primer Sequence	Annealing Temperature (°C)
Promoter-A	F: CCTGAGCTCGCTAGCCTCGAGGCCACCGCTTACAAAGTTCATACCTGTTCCACAG	58
	R: TTGGCCGCCGAGGCCAGATCTGTGTCCACTTGCGTCA	
Promoter-G	F: CCTGAGCTCGCTAGCCTCGAGGCCACCGCTTACAAAGTTCGTACCTGTTCCACAG	58
	R: TTGGCCGCCGAGGCCAGATCTGTGTCCACTTGCGTCA	

Note: F: forward primer; R: reverse primer.

**Table 2:** Impact of *BMP15* variant genotypes on litter size of Gobi short tail sheep.

SNP	Genotype	Number	Litter Size
c.755T>C	TT	179	1.26 ± 0.03
	TC	50	1.34 ± 0.07
g.54291798C>T	CC	211	1.28 ± 0.03
	CT	20	1.15 ± 0.08
g.54292331G>A	GG	201	1.28 ± 0.03
	GA	30	1.20 ± 0.07
g.54292075C>A	CC	204	1.26 ± 0.03
	CA	25	1.32 ± 0.10
g.54288671C>T	CC	27	1.37 ± 0.09
	CT	114	1.30 ± 0.04
	TT	90	1.21 ± 0.04
g.54287453C>T	CC	203	1.27 ± 0.03
	CT	27	1.26 ± 0.09
g.54285159_54285161TTAindel	TTA	114	1.21 ± 0.04 ^a^
	TTA.DEL	102	1.34 ± 0.05 ^b^
	DEL.DEL	15	1.27 ± 0.12 ^ab^

Note: ^a^, ^b^: *p* < 0.05.

**Table 3:** Impact of *BMP15* variant genotypes on the litter size of Ujimqin sheep in this study.

SNP	Genotype	Number	Litter Size
c.755T>C	TT	125	1.19 ± 0.04
	TC	28	1.25 ± 0.08
g.54291460G>A	GG	132	1.18 ± 0.03 ^a^
	GA	21	1.38 ± 0.11 ^b^
g.54292331G>A	GG	133	1.20 ± 0.03
	GA	20	1.25 ± 0.10
g.54292075C>A	CC	114	1.18 ± 0.04
	CA	34	1.29 ± 0.08
g.54288671C>T	CC	31	1.16 ± 0.08 ^ab^
	CT	66	1.28 ± 0.05 ^a^
	TT	56	1.10 ± 0.04 ^b^
g.54285159_54285161TTAindel	TTA.TTA	86	1.14 ± 0.04 ^a^
	TTA.DEL	61	1.31 ± 0.06 ^b^

Note: ^a^, ^b^: *p* < 0.05.

**Table 4:** Impact of *BMP15* variant genotypes on the litter size of the combined Ujimqin sheep population.

SNP	Genotype	Number	Litter Size
c.755T>C	TT	226	1.32 ± 0.03
	TC	43	1.39 ± 0.08
c.1047G>A	GG	254	1.33 ± 0.03
	GA	15	1.33 ± 0.12
g.54291460G>A	GG	238	1.31 ± 0.03 ^A^
	GA	31	1.55 ± 0.09 ^B^
g.54292331G>A	GG	242	1.34 ± 0.03
	GA	27	1.30 ± 0.09
g.54292075C>A	CC	212	1.31 ± 0.03 ^a^
	CA	51	1.47 ± 0.07 ^b^

Note: ^a^, ^b^: *p* < 0.05. ^A^, ^B^: *p* < 0.01.

## Data Availability

Data are contained within the article and supplementary materials.

## Supplementary Materials

The following supporting information can be downloaded at https://www.mdpi.com/article/10.3390/vetsci12030222/s1: Figure S1: Identification of sheep granulosa cells cultured in vitro; Figure S2: The identification of nine variants in ovine BMP15; Figure S3: The prediction of transcription factor binding motifs in partial regions of the promoter sequence, including the g.54291460G>A mutation of the *BMP15* gene. Table S1: PCR primers used for sequencing *BMP15*; Table S2: MassARRAY primers used for the genotyping of nine variants in *BMP15* and one variant in the *FecB* gene; Table S3: Genotypic, allelic frequencies and diversity parameters of ten SNPs in the Gobi short tail sheep and Ujimqin sheep populations; Table S4: Linkage disequilibrium as measured by D’ and r2 among variants in the Gobi short tail sheep population; Table S5: Linkage disequilibrium as measured by D’ and r2 among variants in the Ujimqin sheep of this study; Table S6: Linkage disequilibrium as measured by D’ and r2 among variants in the combined Ujimqin sheep population.
