# Supplementary material for: Variants in BMP15 Gene Affect Promoter Activity and Litter Size in Gobi Short Tail and Ujimqin Sheep
Source: Vet Sci. 2025 Mar 2;12(3):222. doi: 10.3390/vetsci12030222 (PMC11945889; doi:10.3390/vetsci12030222)
Supplement: Supplementary file 1 [file vetsci-12-00222-s001.zip › Figure S2 The identification of nine variants in ovine BMP15.pdf]

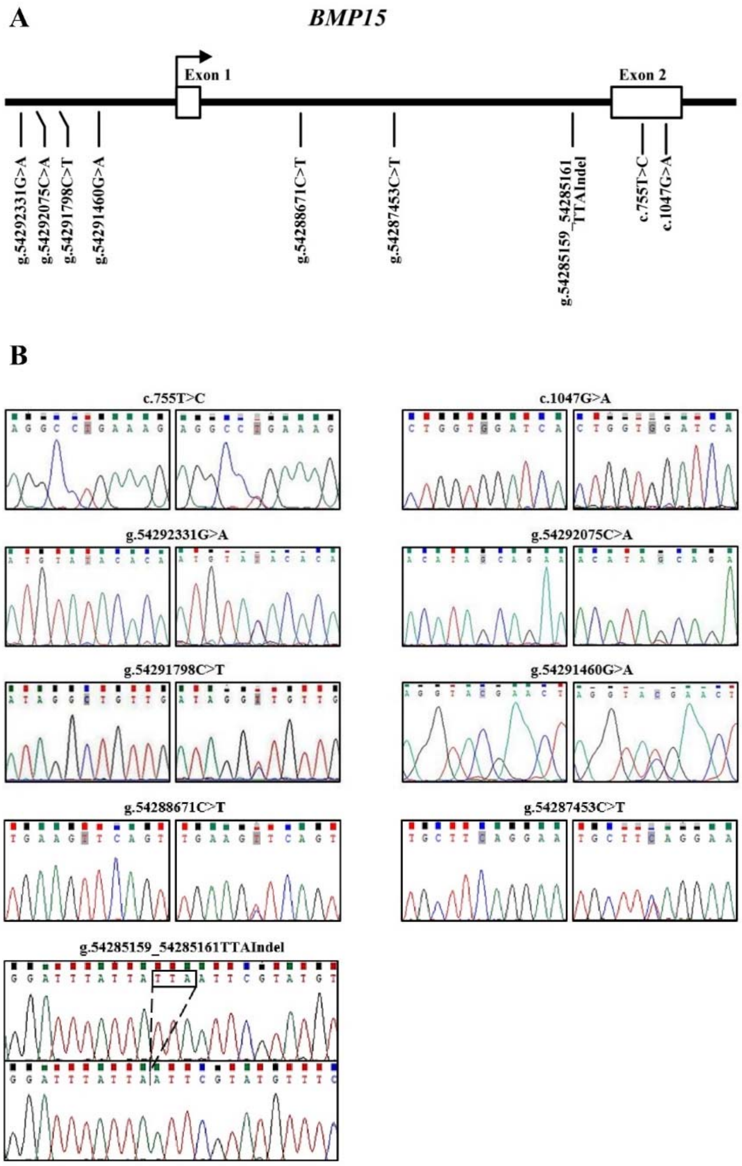


**Figure S2.** The identification of nine variants in ovine *BMP15*. (A) The locations of the nine variants identified in this study are presented. (B) The nucleotide changes in the nine *BMP15* variants are displayed. The variants are mapped to chromosome X in Oar_rambouillet_v2.0 (GenBank accession: NC_056080.1)
