# Supplementary material for: Variants in BMP15 Gene Affect Promoter Activity and Litter Size in Gobi Short Tail and Ujimqin Sheep
Source: Vet Sci. 2025 Mar 2;12(3):222. doi: 10.3390/vetsci12030222 (PMC11945889; doi:10.3390/vetsci12030222)
Supplement: Supplementary file 1 [file vetsci-12-00222-s001.zip › Figure S3 Prediction of transcription factor binding motif in partial regions of promoter sequence including the g.54291460GA mutation of BMP15 gene..pdf]

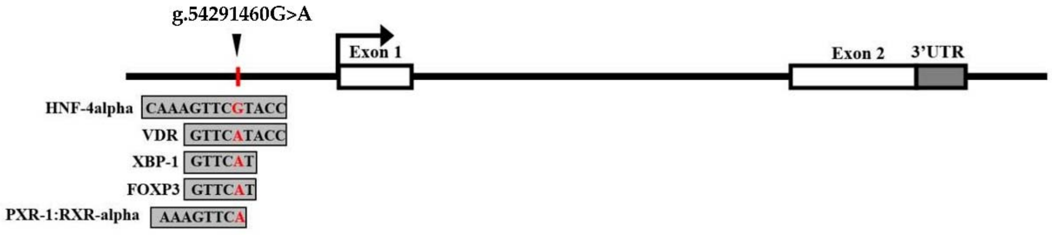

Figure S3. The prediction of transcription factor binding motifs in partial regions of the promoter sequence including the g.54291460G>A mutation of *BMP15* gene.
