# Supplementary material for: Variants in BMP15 Gene Affect Promoter Activity and Litter Size in Gobi Short Tail and Ujimqin Sheep
Source: Vet Sci. 2025 Mar 2;12(3):222. doi: 10.3390/vetsci12030222 (PMC11945889; doi:10.3390/vetsci12030222)
Supplement: Supplementary file 1 [file vetsci-12-00222-s001.zip › Table S1 PCR primers used for sequencing BMP15.pdf]

Table S1. PCR primers used for sequencing *BMP15*.

Primer	Primer Sequence (5'-3')	Target Region	Annealing Tm (°C)	Temperature	Product Dize (bp)
Promoter-1	F: TGAATGGTAGAGCTGGAGTCT<br>R: GTTTCTTCATTTGTACTGCCGG	Promoter	56		612 bp (-2152 ~ -1541 bp promoter) [22]
Promoter-2	F: CCAATTCTAGGTGTGAAGTTGTC<br>R: TTGTAAGCAACGGACCTGTG	Promoter	62		602 bp (-1752 ~ -1150 bp promoter)
Promoter-3	F: CAAGCTGTGGGATAAAACATGT<br>R: TGCTCCCTTACCTTATCCACT	Promoter	56		980 bp (-1296 ~ -317 bp promoter) [22]
Promoter-4	F: CAGCAGCCATACTCACAGAG<br>R: CCTATGTTTCTTCCTCTGGTAGG	Promoter	62		509 bp (-655 ~ -145 bp promoter)
BMP15-1	F: CTGGAGAGCAGGATGGGCAC<br>R: AGCCTTTCAGGACAGCTAAGG	Exon 1	60		1041 (-141 bp promoter + 321 bp exon 1 + 450 bp intron 1)
BMP15-2	F: AACAGACTCTCAGGTGTGAG<br>R: GCAACCCATTTCTTCTTGGA	Intron	60		1279 bp intron [22]
BMP15-3	F: GATTTCTAACCCGGGCTTTC<br>R: GCCAGGCTTGTAATTGCTTT	Intron	62		1365 bp intron [22]
BMP15-4	F: AGATGTATGGGTGGGTGTCTT<br>R: AGCTTGTGTTTCTTCCAGCC	Intron	62		679 bp intron [22]
BMP15-5	F: GGCCATGGTTAGTGACTGGA<br>R: TTCAACATCAGTCCCTCCAA	Intron	62		713 bp intron [22]
BMP15-6	F: GGACTATAAAGGAAGCTGAGCG<br>R: CCTTTCCCTTCTCCAGCGAT	Intron	64		670 bp intron [22]
BMP15-7	F: GCCACTAGGTGTCAGCATCA<br>R: CATACCCCTAGACGGAGAAAAA	Intron	64		686 bp intron [22]
BMP15-8	F: ATTTTGTGGCATCTCCAACC<br>R: ACCCCAAACCGTCTAGATCC	Exon 2	64		1684bp (576 bp intron + 857 bp exon 2 + 251 bp 3 'UTR) [22]

Note. F: forward primer; R: reverse primer; and UTR: untranslated region. RefSeq: NC_056080.1(Start at 54283635 and end at 54290315); Promoter fragment is located in front of the transcription start site.
