# Supplementary material for: Variants in BMP15 Gene Affect Promoter Activity and Litter Size in Gobi Short Tail and Ujimqin Sheep
Source: Vet Sci. 2025 Mar 2;12(3):222. doi: 10.3390/vetsci12030222 (PMC11945889; doi:10.3390/vetsci12030222)
Supplement: Supplementary file 1 [file vetsci-12-00222-s001.zip › Table S2 MassARRAY primers used for genotyping of nine variants in BMP15 and one variant in Fec B gene..pdf]

Table S2. MassARRAY primers used for the genotyping of nine variants in *BMP15* and one variant in *Fec B* gene.

Mutation	Primer Sequence (5'-3')	Annealing Temperature (°C)
g.54292331G>A	F: ACGTTGGATGACACTTTACATCCAGCCCAC<br>R: ACGTTGGATGAGGAAATGCTTACTTCAGG<br>E: ACACATGTGCATGTA	53.7
g.54292075C>T	F: ACGTTGGATGGCATTCTACATCCTAACCTG<br>R: ACGTTGGATGGCCACATAAGACAACTTCAC<br>E: CTAACATTAGACTTCTG	47.7
g.54291798C>T	F: ACGTTGGATGTCTTGTTAGAGGTCACCAAC<br>R: ACGTTGGATGAGATATGGGTCCCCTAGGTC<br>E: CTCTGTCACCAACTAATAGG	51.9
g.54291460G>A	F: ACGTTGGATGAGGTGGGACAGAAAGTCAAG<br>R: ACGTTGGATGACAATCCAAACACAGGTCCG<br>E: GCTCTGTGGAACAGGTA	60.4
g.54288671C>T	F: ACGTTGGATGACTGCTCCACTATGGAATAC<br>R: ACGTTGGATGCGGGTTAGAAATCCAGCTC<br>E: GGGTGAAGAAAATGAAG	51
g.54287453C>T	F: ACGTTGGATGCTTTCAGACCCTGACATCAC<br>R: ACGTTGGATGAAGTTCTGAGCAGACCCTTC<br>E: GCATCACATATTTCCT	47.2
g.54285159_54285161TTAIndel	F: ACGTTGGATGGAGGATCATTGAAACATACG<br>R: ACGTTGGATGTTGGACTGTCACCCTAAAGC<br>E: GGTTAAACATACGAATTAATAA	48
c.755T>C	F: ACGTTGGATGCTCAGAGTGTTCAGAAGACC<br>R: ACGTTGGATGCTCAAGAGAAGAGAAGGGTC<br>E: GCACCCCTCTCCCTAAAGGCC	62.5

c.1047G>A	F: ACGTTGGATGCTCCCAATCATGCCATCATC<br>R: ACGTTGGATGTATAAGGGACACAGGAAGGC<br>E: CCCCCCCTTGTCAGTGAGCTGGT	64
FecB	F: ACGTTGGATGCCAAGATGTTTTCATGCCTC<br>R: ACGTTGGATGTTCTTCACTACAGAGGAGGC<br>E: CCTCATCAACACCGTC	46.7

Note: F: forward primer. R: reverse primer. E: extended primer.
