# Supplementary material for: Variants in BMP15 Gene Affect Promoter Activity and Litter Size in Gobi Short Tail and Ujimqin Sheep
Source: Vet Sci. 2025 Mar 2;12(3):222. doi: 10.3390/vetsci12030222 (PMC11945889; doi:10.3390/vetsci12030222)
Supplement: Supplementary file 1 [file vetsci-12-00222-s001.zip › Table S3 Genotypic, allelic frequencies and diversity parameters of ten SNPs in the Gobi short tail sheep and Ujimqin sheep populations..pdf]

Table S3. Genotypic, allelic frequencies and diversity parameters of ten SNPs in the Gobi short tail sheep and Ujimqin sheep populations.

Mutation/Breed	Genotype Frequency			Allele Frequency		Diversity parameter				
						H_o	H_e	n_e	PIC	χ^2 (HWE[2])
c.755T>C	TT	TC	CC	T	C					
GB	0.775	0.216	0.009	0.883	0.117	0.794	0.206	1.260	0.185	0.543
UM	0.817	0.183	0.009	0.908	0.092	0.834	0.166	1.199	0.153	1.552
c.1047G>A	GG	GA	AA	G	A					
GB	0.961	0.039	0.000	0.981	0.019	0.962	0.038	1.040	0.037	0.091
UM	0.948	0.052	0.000	0.974	0.026	0.949	0.051	1.054	0.049	0.110
g.54291460G>A	GG	GA	AA	G	A					
GB	0.996	0.004	0.000	0.998	0.002	0.996	0.004	1.004	0.004	0.001
UM	0.863	0.137	0.000	0.931	0.069	0.872	0.128	1.147	0.120	0.831
g.54291798C>T	CC	CT	TT	C	T					
GB	0.913	0.087	0.000	0.957	0.043	0.917	0.083	1.090	0.079	0.473
UM	0.967	0.033	0.000	0.984	0.016	0.968	0.032	1.033	0.031	0.042
g.54292331G>A	GG	GA	AA	G	A					
GB	0.870	0.130	0.000	0.935	0.065	0.879	0.121	1.138	0.114	1.114
UM	0.869	0.131	0.000	0.935	0.065	0.878	0.122	1.139	0.114	0.748
g.54292075C>A	CC	CA	AA	C	A					
GB	0.883	0.108	0.009	0.937	0.063	0.882	0.118	1.133	0.111	1.486
UM	0.745	0.222	0.033	0.856	0.144	0.754	0.246	1.327	0.216	1.455
g.54288671C>T	CC	CT	TT	C	T					
GB	0.117	0.494	0.390	0.364	0.636	0.537	0.463	1.862	0.356	1.016
UM	0.203	0.431	0.366	0.418	0.582	0.513	0.487	1.948	0.368	1.974
g.54287453C>T	CC	CT	TT	C	T					
GB	0.879	0.117	0.004	0.937	0.063	0.882	0.118	1.133	0.111	0.010

UM	1.000	0.000	0.000	1.000	0.000	1.000	0.000	1.000	0.000	-
g.54285159_54285161DEL.TTA	TTA	DEL.TTA	DEL.DEL	TTA	DEL					
GB	0.494	0.442	0.065	0.714	0.286	0.592	0.408	1.690	0.325	1.546
UM	0.562	0.399	0.039	0.761	0.239	0.637	0.363	1.571	0.298	1.452
c.746A>G(*FecB*)	AA	AG	GG	A	G					
GB	1.000	0.000	0.000	1.000	0.000	1.000	0.000	0.000	0.000	-
UM	1.000	0.000	0.000	1.000	0.000	1.000	0.000	0.000	0.000	-

Note: SE: Standard Error. H_o: Observed Heterozygosity. H_e: Expected Heterozygosity. n_e: Effective Allele Numbers. PIC: Polymorphism Information Content. HWE: Hardy-Weinberg Equilibrium. GB: Gobi short tail sheep. UM: Ujimqin sheep. These sheep were the data in this study.
