# Supplementary material for: Variants in BMP15 Gene Affect Promoter Activity and Litter Size in Gobi Short Tail and Ujimqin Sheep
Source: Vet Sci. 2025 Mar 2;12(3):222. doi: 10.3390/vetsci12030222 (PMC11945889; doi:10.3390/vetsci12030222)
Supplement: Supplementary file 1 [file vetsci-12-00222-s001.zip › Table S4 Linkage disequilibrium as measured by D' and r2 among variants in the Gobi short tail sheep population.pdf]

**Table S4.** Linkage disequilibrium as measured by D' and $r^2$ among variants in the Gobi short tail sheep population.

| | g.755 T>C | c.1047 G>A | Indel-3bp | g.54287453 G>T | g.54288671 C>T | g.54291460 G>A | g.54291798 C>T | g.54292075 C>A |
|---|---|---|---|---|---|---|---|---|
| c.1047G>A | D' = 1.000 $r^2$ = 0.003 | | | | | | | |
| Indel-3bp | D' = 0.968 $r^2$ = 0.310 | D' =1.000 $r^2$ = 0.008 | | | | | | |
| g.54287453C>T | D' = 1.000 $r^2$ = 0.009 | D' = 0.100 $r^2$ = 0.001 | D' = 1.000 $r^2$ = 0.027 | | | | | |
| g.54288671C>T | D' = 0.961 $r^2$ = 0.214 | D' = 1.000 $r^2$ = 0.011 | D' = 0.920 $r^2$ = 0.593 | D' = 1.000 $r^2$ = 0.038 | | | | |
| g.54291460G>A | D' = 1.000 $r^2$ = 0.000 | D' = 1.000 $r^2$ = 0.000 | D' = 1.000 $r^2$ = 0.001 | D' = 1.000 $r^2$ = 0.000 | D' = 1.000 $r^2$ = 0.004 | | | |
| g.54291798C>T | D' = 0.181 $r^2$ = 0.011 | D' = 1.000 $r^2$ = 0.001 | D' = 0.141 $r^2$ = 0.000 | D' = 0.029 $r^2$ = 0.001 | D' = 0.101 $r^2$ = 0.001 | D' = 1.000 $r^2$ = 0.000 | | |
| g.54292075C>A | D' = 0.669 $r^2$ = 0.004 | D' = 1.000 $r^2$ = 0.000 | D' = 1.000 $r^2$ = 0.027 | D' = 1.000 $r^2$ = 0.004 | D' = 1.000 $r^2$ = 0.117 | D' = 1.000 $r^2$ = 0.032 | D' = 0.029 $r^2$ = 0.001 | |
| g.54292331G>A | D' = 1.000 $r^2$ = 0.009 | D' = 0.318 $r^2$ = 0.000 | D' = 0.751 $r^2$ = 0.098 | D' = 1.000 $r^2$ = 0.005 | D' = 1.000 $r^2$ = 0.122 | D' = 1.000 $r^2$ = 0.000 | D' = 1.000 $r^2$ = 0.003 | D' = 1.000 $r^2$ = 0.005 |

Note: Indel-3-bp: g.54285159_54285161TTAindel.
