# Supplementary material for: Variants in BMP15 Gene Affect Promoter Activity and Litter Size in Gobi Short Tail and Ujimqin Sheep
Source: Vet Sci. 2025 Mar 2;12(3):222. doi: 10.3390/vetsci12030222 (PMC11945889; doi:10.3390/vetsci12030222)
Supplement: Supplementary file 1 [file vetsci-12-00222-s001.zip › Table S5. Linkage disequilibrium as measured by D' and r2 among variants in the Ujimqin sheep of this study.pdf]

Table S5. Linkage disequilibrium as measured by D' and r^2 among variants in the Ujimqin sheep of this study.

	**c.755 T>C**	**c.1047 G>A**	**Indel-3bp**	**g.54288671 C>T**	**g.54291460 G>A**	**g.54291798 C>T**	**g.54292075 C>A**
c.1047G>A	D' = 1.000 r^2 = 0.003						
Indel-3bp	D' = 1.000 r^2 = 0.321	D' = 1.000 r^2 = 0.008					
g.54288671C>T	D' = 0.644 r^2 = 0.058	D' = 1.000 r^2 = 0.019	D' = 1.000 r^2 = 0.271				
g.54291460G>A	D' = 1.000 r^2 = 0.007	D' = 0.199 r^2 = 0.000	D' = 1.000 r^2 = 0.023	D' = 1.000 r^2 = 0.102			
g.54291798C>T	D' = 1.000 r^2 = 0.002	D' = 1.000 r^2 = 0.000	D' = 1.000 r^2 = 0.005	D' = 1.000 r^2 = 0.012	D' = 0.074 r^2 = 0.001		
g.54292075C>A	D' = 1.000 r^2 = 0.017	D' = 0.327 r^2 = 0.000	D' = 0.751 r^2 = 0.030	D' = 1.000 r^2 = 0.234	D' = 1.000 r^2 = 0.439	D' = 0.752 r^2 = 0.002	
g.54292331G>A	D' = 0.037 r^2 = 0.001	D' = 1.000 r^2 = 0.002	D' = 0.626 r^2 = 0.083	D' = 1.000 r^2 = 0.092	D' = 1.000 r^2 = 0.005	D' = 1.000 r^2 = 0.001	D' = 0.226 r^2 = 0.001

Note: Indel-3-bp: g.54285159_54285161TTAindel.
