# Supplementary material for: Variants in BMP15 Gene Affect Promoter Activity and Litter Size in Gobi Short Tail and Ujimqin Sheep
Source: Vet Sci. 2025 Mar 2;12(3):222. doi: 10.3390/vetsci12030222 (PMC11945889; doi:10.3390/vetsci12030222)
Supplement: Supplementary file 1 [file vetsci-12-00222-s001.zip › Table S6. Linkage disequilibrium as measured by D' and r2 among variants in the combined Ujimqin sheep population..pdf]

Table S6. Linkage disequilibrium as measured by D' and r^2 among variants in the combined Ujimqin sheep population.

	c.755 T>C	c.1047 G>A	g.54291460 G>A	g.54291798 C>T	g.54292075 C>A
c.1047G>A	D' = 1.000 r^2 = 0.002				
g.54291460G>A	D' = 0.939 r^2 = 0.005	D' = 0.929 r^2 = 0.002			
g.54291798C>T	D' = 1.000 r^2 = 0.001	D' = 1.000 r^2 = 0.000	D' = 0.059 r^2 = 0.001		
g.54292075C>A	D' = 1.000 r^2 = 0.012	D' = 1.000 r^2 = 0.004	D' = 1.000 r^2 = 0.461	D' = 0.131 r^2 = 0.001	
g.54292331G>A	D' = 0.088 r^2 = 0.002	D' = 0.417 r^2 = 0.098	D' = 1.000 r^2 = 0.003	D' = 1.000 r^2 = 0.001	D' = 0.444 r^2 = 0.001
