# Supplementary figures and images for: Variants in BMP15 Gene Affect Promoter Activity and Litter Size in Gobi Short Tail and Ujimqin Sheep
Source: Vet Sci. 2025 Mar 2;12(3):222. doi: 10.3390/vetsci12030222 (PMC11945889; doi:10.3390/vetsci12030222)

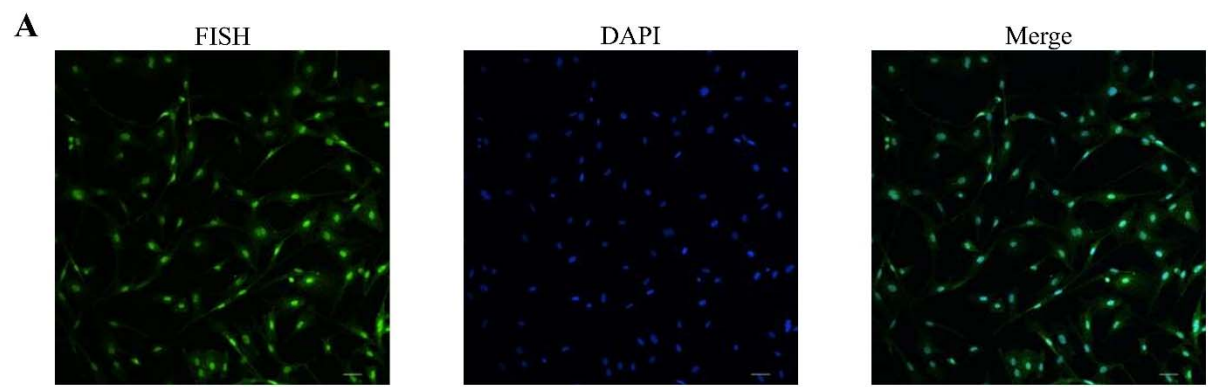

**Figure S1.** Identification of sheep granulosa cells cultured in vitro.

Supplement: Supplementary file 1 [file vetsci-12-00222-s001.zip › Figure S1 Identification of sheep granulosa cells cultured in vitro.pdf]
